# Comparative evaluation of antimicrobial activity of human granulysin, bovine and porcine NK-lysins against Shiga toxin-producing *Escherichia coli* O157:H7

**DOI:** 10.1371/journal.pone.0292234

**Published:** 2023-09-28

**Authors:** Erika N. Biernbaum, Rohana P. Dassanayake, Eric M. Nicholson, Indira T. Kudva

**Affiliations:** 1 Food Safety and Enteric Pathogens Research Unit, National Animal Disease Center, Agricultural Research Service, U.S. Department of Agriculture, Ames, Iowa, United States of America; 2 Oak Ridge Institute for Science and Education (ORISE), ARS Research Participation Program, Oak Ridge, Tennessee, United States of America; 3 Ruminant Diseases and Immunology Research Unit, National Animal Disease Center, Agricultural Research Service, U.S. Department of Agriculture, Ames, Iowa, United States of America; 4 Virus and Prion Research Unit, National Animal Disease Center, Agricultural Research Service, U.S. Department of Agriculture, Ames, Iowa, United States of America; University of Jeddah, SAUDI ARABIA

## Abstract

Shiga toxin-producing *Escherichia coli* (STEC) O157:H7 (O157) is a foodborne pathogen causing human disease ranging from hemorrhagic colitis and hemolytic uremic syndrome to kidney failure, while remaining harmless to cattle, its primary reservoir. The severity of the human disease associated mainly with Shiga toxin production and a global emergence of antibiotic resistant STEC highlights the need for effective non-antibiotic, pre-harvest strategies to reduce O157 in cattle, the principal source of human infection. Towards this goal three synthetic antimicrobial peptides (AMPs): human granulysin (hGRNL), bovine NK-lysin (bNK2A), and porcine NK-lysin (pNKL), were tested *in vitro* against O157 isolates. As expected, circular dichroism spectroscopy findings were consistent with a predominantly α-helical conformation for all three AMPs in an environment mimicking bacterial outer surface or liposaccharides. The minimum inhibitory concentrations (MIC) and minimum bactericidal concentrations of hGRNL (200 μM), bNK2A (12.5 μM against strain 86–24 and 25 μM against EDL933), and pNKL (6.25 μM) were determined using the Clinical and Laboratory Standards Institute broth microdilution method in Müeller-Hinton broth (cation-adjusted). The bNK2A and pNKL AMPs did not induce Shiga toxin expression in O157 at MIC, as there was a significant decrease or no change in toxin expression following 4- or 20 h incubation with the AMPs; bNK2A *p* <0.0001 (4 h) and *p* = 0.4831 (20 h); pNKL *p* <0.0001 (4 h) and *p =* 0.0001 (20 h). Propidium iodide uptake assay revealed faster O157 membrane damage or killing kinetics with bNK2A and pNKL compared to hGRNL. Nonetheless, transmission electron microscopy demonstrated that all three AMPs mediated damage to O157 membranes. In contrast, the three AMPs showed minimal cytotoxicity (<2%) against cattle red blood cells at tested concentrations (0.39–50 μM). Overall, our results demonstrate the potential for bNK2A and pNKL to be further developed into novel non-antibiotic agents to reduce O157 shedding in cattle.

## Introduction

Shiga toxin-producing *Escherichia coli* (STEC), first isolated in 1982, are responsible for causing ~265,000 human illnesses in the United States annually [1] and 2.8 million globally [2]. The serotype O157:H7 (O157) is implicated most often in STEC infections and hence, the most-researched STEC. However, non-O157 STEC can also cause illness in humans and is likely that cases have been under-reported, as identification of non-O157 infections is more elaborate than that for O157 and testing practices are oftentimes oriented towards O157 detection, especially in the past [3]. STEC are foodborne pathogens, transmitted *via* the fecal-oral route or by encountering infected humans or animals [3–7]. Typical infections are characterized by watery or bloody diarrhea that can progress to hemolytic uremic syndrome, thrombocytopenic purpura, and kidney failure due to various virulence factors like Shiga toxins (*stx1* and *stx2*) [4, 8–11].

Cattle are the main reservoirs of STEC and are asymptomatic carriers of the human pathogen due to a lack of Gb_3_ receptors in the gastrointestinal tract for Shiga toxin uptake [12]. STEC preferentially colonize the rectoanal junction in cattle [13], greatly impacting the load of STEC in feces and transmission among cattle. This can lead to increased shedding of STEC in the environment and fecal contamination of food, a major risk factor for human infection. Currently there are no effective pre-harvest control strategies for STEC in reservoirs, and treatment options for patients are often limited to supportive therapies, highlighting the need for preventative methods.

Antimicrobial peptides (AMPs) or host defense peptide (HDP) are a diverse group of evolutionary conserved molecules produced by all living organisms and play a vital role in the innate immune response [14]. AMPs show remarkable structural and functional diversity. AMPs can be subdivided into several subgroups based on the amino acid composition and structure; however, cationic (α-helical and β-sheet) AMPs form the prominent subgroup [14, 15]. Positively-charged cationic AMPs interact with negatively-charged microbial outer- (lipopolysaccharides of Gram negative and peptidoglycans of Gram positive) and inner plasma-membranes by electrostatic attraction [15, 16]. Such electrostatic attraction and binding of cationic AMPs to bacteria were previously demonstrated by zeta sizer and transmission electron microscopy, respectively [16].

Antimicrobial proteins human granulysin, bovine and porcine NK-lysins are structurally and functionally similar to each other and have been very effective against both Gram-positive and Gram-negative bacteria [17–19]. They are produced by cytotoxic T lymphocytes and natural killer cells [20–22]. All three AMPs are positively charged (or cationic) and structurally related to saposin-like protein (SAPLIP) family of lipid binding proteins and thus contain the saposin B domain [18, 23]. Unlike human and swine genomes, cattle genome has four functional NK-lysin genes and show tissue-specific expression patterns [24]. Based on the success of bovine NK-lysin peptides against *Salmonella* [16], another foodborne pathogen, bovine NK-lysin peptide bNK2A along with human granulysin (hGRNL), and porcine NK-lysins (pNKL) were selected against two O157 strains as potential anti-STEC agents. Peptides corresponding to the functional region helix2-loop-helix3 of hGRNL, bNK2A, and pNKL [16, 19, 20] were chemically synthesized and their *in vitro* efficacy against O157 strains evaluated.

## Materials and methods

### Peptide synthesis and amino acid analysis

Human granulysin (hGRNL: QRSVSNAATRVCRTGRSRWRDVCRNFMRR) [18, 19], bovine NK-lysin NK2A (bNK2A: TVIEVASKMCSKMRLLKGLCKSITKRFLRR) [17, 20], and porcine NK-lysin (pNKL: TVTQAASRVCDKMKILRGVCKKIMRTFLRR) [19, 22, 25] peptides, corresponding to the functional region helix2-loop-helix3, were synthesized by 9-fluorenylmethyl chloroformate (FMOC) solid-phase peptide synthesis chemistry, and supplied as trifluoroacetate salt (Peptide 2.0 Inc., Chantilly, VA). The purity of peptides was over 95% as assessed by reverse phase high performance-liquid chromatography (RP-HPLC) on an analytical Agela C_18_ column and the identity (and mass) of the peptides was confirmed by mass-spectrometry (Peptide 2.0 Inc). To enhance stability and prevent enzymatic degradation, peptide ends were modified by N-terminal acetylation and C-terminal amidation. Lyophilized peptides were dissolved in Gibco™ Dulbecco’s phosphate-buffered saline (DPBS, pH 7.2, calcium and magnesium free; Life Technologies Ltd., Paisley, PA), or sodium phosphate buffer (20 mM, NaPB, pH 7.4; Mallinckrodt Baker, Inc., Phillipsburg, NJ), aliquoted, and stored at -80°C until used. All the experiments were performed with the same batch of peptides. Peptide concentrations were determined by quantitative amino acid analysis at the Center for Biotechnology, University of Nebraska-Lincoln, Lincoln, NE.

### Circular dichroism (CD) spectrophotometry analysis

CD analyses of hGRNL, bNK2A, and pNKL peptides were performed as described previously, but with minor modifications [26, 27]. Each peptide (final concentration = 20 μM) dissolved in NaPB was mixed with lipopolysaccharides (LPS, final concentration = 0.1% (w/v)), double-distilled water (to adjust the final NaPB concentration to 10 mM) and placed in a 1 mm path-length quartz cuvette (final volume = 300 μl). The secondary structures of the peptides were then evaluated using a Jasco J-815 CD spectrophotometer (Jasco, Easton, MA). Measurements were taken every 0.2 nm from 250 to 190 nm with six accumulations at room temperature using automated baseline correction. Following background subtraction, raw data in millidegrees was converted to mean residue ellipticity (MRE). Spectra in MRE were imported into Dichroweb online data analysis (http://dichroweb.cryst.bbk.ac.uk/html/home.shtml) [28] using Contin-LL analysis [29] with data set 4 [30, 31].

### Hemolytic activity assay

The hemolytic activity of the three AMPs was evaluated using cattle red blood cells (RBCs) as described previously [26]. Different concentrations of the AMPs (two-fold dilution 50 to 0.39 μM) (L_Exp_) were incubated with RBCs at 37°C in a humidified atmosphere for 60 min and released hemoglobin was estimated using a microplate reader by measuring the absorbance at 405 nm wavelength. RBCs incubated with Triton X-100 (0.1% (v/v) final concentration, L_Tx100_) was used as a positive control (100% lysis) and RBCs incubated with DPBS (L_0_) was used as a negative control. The percentage of hemolysis was calculated by using the following formula (L_Exp_-L_0_)/L_Tx100_-L_0_) × 100. Mean and standard error of mean (SEM) for percent hemolysis were calculated from RBCs prepared from two cattle.

### O157 strains and culture conditions

The following O157 strains were used in peptide susceptibility assays: (i) EDL933 (ATCC 43895; *stx1*^*+*^, *stx2*^*+*^, *eae*^*+*^, *hlyA*^*+*^; American Type Culture Collection/ATCC, Manassas, VA) and (ii) 86–24 (NADC 6103; *stx2*^*+*^, *eae*^*+*^). O157 strains EDL933 and 86–24 displayed similar results in initial peptide susceptibility assays, and due to EDL933 having both *stx* genes, only strain EDL933 was used in subsequent assays. Bacterial freezer stocks were streaked individually on Luria-Bertani (LB) Lennox agar (Sigma-Aldrich, St. Louis, MO) and incubated overnight at 37˚C. Bacterial cultures were prepared by growing each isolate from a single colony in 5 mL LB broth (Sigma-Aldrich) per assay for 20 h at 37˚C with constant shaking at 150 rpm. Assay isolates were streaked on Difco sorbitol MacConkey agar (BD Biosciences, Franklin Lakes, NJ) containing 4-methylumbelliferyl-β-d-glucuronide (100 mg/L; Sigma-Aldrich) (SMAC-MUG) or SMAC-MUG supplemented with cefixime (50 μg/L), potassium tellurite (2.5 mg/L), and vancomycin (40 mg/L) (SMAC-CTMV) agar [32, 33]. Plates were read after overnight incubation at 37˚C and colonies that did not ferment sorbitol or utilize 4-methylumbelliferyl-β-d-glucuronide (non-fluorescent under UV light) were confirmed to be O157 *via* latex agglutination (*E*. *coli* O157 latex, Oxoid Diagnostic Reagents, Oxoid Ltd., Hampshire, UK) [32, 33].

### Susceptibility assays

#### O157-peptide susceptibility assay in Müeller-Hinton II broth (MHB)

Trial assays were set in duplicate using Clinical and Laboratory Standards Institute (CLSI) guidelines [34] as follows. Overnight cultures of O157 strain EDL933 were diluted in cation-adjusted (calcium and magnesium ions) 2x BD BBL™ MHB (Becton, Dickinson and Co., Spark, MD) to a concentration of ~ 1×10^6^ CFU/mL. Fifty microliters of culture (~5×10^5^ CFU/ well) were transferred to 96-well flat-bottom polystyrene plates (Falcon #351172; Corning, NY) within 15 min of preparation, and 50 μL of hGRNL, bNK2A, or pNKL peptides in filter-sterilized Gibco™ DPBS (Life Technologies Ltd.) were added to final concentrations of 0.391, 0.782, 1.56, 3.13, 6.26, 12.5, 25, and 50 μM. Additional concentrations of hGRNL (50, 100, 200, and 400 μM) were also tested in later assays when no inhibition of bacterial growth was observed with the -80°C stored peptides, at the concentrations used in the first two trials. The plates were covered with lids, placed in plastic bags with a moist tissue for humidity, and incubated without aeration for 20 h at 37˚C. Bacteria incubated with antibiotics tetracycline or ciprofloxacin and 2X MHB without AMP were used as positive and negative controls for the assay, respectively. Tetracycline concentrations tested were 0.125, 0.25, 0.5, 1, 2, 4, 8, and 16 μg/mL and ciprofloxacin concentrations tested were 0.016, 0.032, 0.064, 0.125, 0.25, 0.5, 1, 2, and 5 μg/mL.

After 20 h incubation, the minimum inhibitory concentration (MIC: the lowest concentration of an antimicrobial agent that completely and visibly inhibits bacterial growth) was determined by **(i)** the unaided eye and **(ii)** measuring the optical density (OD_600nm_) on a Synergy HT microplate reader (BioTek Instruments, Winooski, VT) with medium shaking for 5 sec. In addition to the MIC, the minimum bactericidal concentration (MBC; the lowest concentration of an antimicrobial agent that results in a 3-log (>99.9% killing) reduction in bacterial viability) was also determined. Cultures from the wells of at least two of the more concentrated peptides compared to the MIC, the MIC, and one peptide concentration below the MIC were diluted in DPBS and 10 μL drops were plated in triplicate on LB agar plates. Plates were incubated overnight at 37˚C or at room temperature and bacterial colonies were enumerated in 1 or 2 days, respectively. The MBC was calculated using the following equation: 100 –[(average CFU/mL bacteria post peptide incubation × 100) / average CFU/mL bacteria in negative control]. The susceptibility assays for O157 strains were repeated three times in triplicate wells.

O157 strain 86–24 was also tested in the same fashion as above and produced similar susceptibility results as EDL933. Therefore, the more prototypical O157 strain EDL933, containing both *stx* genes, was used in subsequent assays.

#### O157-hGRNL comparative susceptibility assays in MHB and NaPB

hGRNL was not as effective as pNKL and bNK2A in MHB under the same conditions (20 h incubation at 37°C with aeration) in controlling growth of O157; therefore, we sought to test if the divalent ions (Ca^2+^ and Mg^2+^) present in MHB interfere with the antimicrobial properties of the hGRNL peptide. We conducted a comparative susceptibility assay between MHB and NaPB with O157 strain EDL933 to test this, as previously described with the following modifications [17, 35]. Overnight bacterial cultures were washed (10 mM NaPB, pH 7.4) or used directly (2X MHB) and diluted in respective media to ~1×10^6^ CFU/mL. Within 15 min of inoculum preparation, 50 μL of culture (~5x10^5^ CFU/mL per well) were transferred to 96-well flat-bottom polystyrene plates and 50 μL hGRNL at final concentrations of 1.56, 3.13, 6.26, 12.5, 25, 50, 100, or 200 μM in either MHB or NaPB were added. hGRNL concentrations in NaPB were further reduced to 0.195, 0.39, 0.78, 1.56, 3.13, 6.26, 12.5, and 25 μM in the second trial due to complete inhibition of O157 strain EDL933 growth at the higher concentrations; the hGRNL concentrations in MHB remained unchanged. The plates were covered with lids, placed in plastic bags, and incubated for 3 h at 37˚C with shaking at 100 rpm. Bacteria incubated with ciprofloxacin (0.016, 0.032, 0.064, 0.128, 0.256, 0.512, 1, and 2 μg/mL) and MHB or NaPB without AMP were used as positive and negative controls, respectively. The killing percentage was determined as described above, except each well was diluted and plated. The comparative susceptibility assays were repeated twice, in duplicate wells.

#### O157-peptide susceptibility assays in NaPB

To see if the differences observed with the antimicrobial effects of the hGRNL peptide in different media also occurs with the bNK2A, and pNKL peptides, we set up additional susceptibility assays in NaPB, similar to the comparative assay above. Briefly, overnight cultures of O157 strain EDL933 were washed and diluted in NaPB to ~1×10^6^ CFU/mL. Fifty microliters of bacterial culture were transferred to 96-well flat-bottom polystyrene plates and 50 μL of pNKL or bNK2A peptides in NaPB were added at final concentrations of 0.195, 0.39, 0.78, 1.56, 3.13, 6.26, 12.5, and 25 μM. Plates were covered with lids, placed in plastic bags, and incubated for 3 h at 37˚C with shaking at 100 rpm. Bacteria with no AMP and NaPB were used as controls and the killing percentages were determined as described above. The susceptibility assays were repeated twice in duplicate wells.

### Propidium iodide (PI) uptake assay

The killing kinetics of peptide-induced damage to O157 cell membranes were evaluated using the PI uptake assay as described previously [36]. Briefly, an overnight culture of O157 strain EDL933 was grown in 10 mL LB broth at 37˚C with shaking at 150 rpm. The culture was centrifuged (5000 rpm for 30 min), washed once in NaPB, and diluted to ~2–4×10^9^ CFU/mL in NaPB. Ninety-five microliters of bacterial suspension were transferred to clear-bottom black-walled 96-well polystyrene plates (Thermo #265301; ThermoFisher Scientific, Inc., Waltham, MA), mixed with 5 μL PI (Thermo #L13152; final concentration of 3 μM dissolved in NaPB), and incubated at 37˚C in the dark for 5 min. One hundred μL of hGRNL, bNK2A, or pNKL peptides (all at final concentrations of 0.80, 1.56, 3.13, 6.25, 12.5, 25, and 50 μM) were added, mixed, and the PI fluorescent intensities in each well were continuously measured for 10 min (30 sec intervals, flex mode) using a FlexStation 3 multi-mode (fluorescent) microplate reader (Molecular Devices, LLC., San Jose, CA). The excitation and emission wavelengths for PI were set to 530 nm and 620 nm, respectively. O157 strain EDL933 incubated with NaPB was used as a negative control. Experiments were repeated three times per each peptide.

### Transmission electron microscopy (TEM)

Morphological changes of *E*. *coli* O157 strain EDL933 following incubation with hGRNL, bNK2A, or pNKL peptides were examined by TEM. Overnight cultures of O157 strain EDL933 were centrifuged (5000 rpm for 30 min) and diluted to ~2×10^9^ CFU/mL in NaPB. Fifty microliters of bacterial suspension were placed in 96-well flat-bottom polystyrene plates (Falcon #351172) and hGRNL, bNK2A, pNKL (20 μM final concentration) or NaPB were added and incubated at 37˚C for 15 (or 30 min) with continuous shaking. The bacterial suspensions were mixed with 120 μL of 3% glutaraldehyde in 0.1 M cacodylate buffer (pH 7.4) and processed for TEM as described previously [26]. Ultrathin sections were stained with uranyl acetate and lead citrate and, examined with a TEM (FEI Tecnai G2 Biotwin; FEI Co., Hillsboro, OR) and images were captured with Advanced Microscopy Technologies (AMT Inc., Danvers, MN) imaging camera. The final figures were prepared using Adobe Photoshop Elements 11 (Adobe Systems Inc., San Jose, CA).

### Immunoassay for Shiga toxin expression

An Stx-detecting enzyme-linked immunosorbent assay (ELISA) was used to assess whether the AMPs induce Shiga toxin production in O157 after treatment. The Premier^®^ EHEC kit (Meridian Bioscience, Cincinnati, OH; https://www.meridianbioscience.com/diagnostics/disease-areas/gastrointestinal/e-coli/premier-ehec/), which detects Shiga toxins I and II, was utilized per the manufacturer’s instructions, with a few modifications. Briefly, AMP susceptibility assays in 2X MHB were set up as described above (*O157-peptide susceptibility assays in Müeller-Hinton broth (MHB)*), using the respective concentrations of hGRNL (200–800 μM), bNK2A (25–50 μM), and pNKL (6.25–12.5 μM) peptides. O157 strain EDL933 overnight cultures were diluted to ~1×10^6^ CFU/mL and incubated with the peptides for either 4 h (with shaking) or 20 h (static) to evaluate bacteria at the log- and stationary- growth phase. A bacterial culture with no peptide was included as a control. The log- and stationary-phase post-assay bacterial cultures were immediately collected and centrifuged for 3 min at 13,000 rpm at 4°C and the supernatants used for plate counts and ELISA set-up. Supernatants were either undiluted or diluted in sample diluent (Premier^®^ EHEC kit) as follows: post- 4 h doubling dilutions up to 1:256 and post-20 h dilutions of 1:5, 1:50, 1:500, and 1:1000. One-hundred microliters of undiluted or diluted sample was used to set up the ELISA, in duplicate, and plates were subsequently read spectrophotometrically at 450 nm on a Synergy HT microplate reader (BioTek Instruments) with medium shaking for 5 sec. Samples were considered positive for the presence of Shiga toxins when the OD_450nm_ was ≥0.180 (Premier^®^ EHEC kit).

### Statistics

EHEC-ELISA OD_450nm_ data was evaluated using descriptive statistics and unpaired *t*-tests for statistical significance of O157 cultures treated with AMP versus O157 cultures without AMP; *p* < 0.05 was considered significant (GraphPad Prism version 8.4.3, GraphPad Software, San Diego, CA).

## Results

### Secondary structures of granulysin and NK-lysins

**Fig 1:**
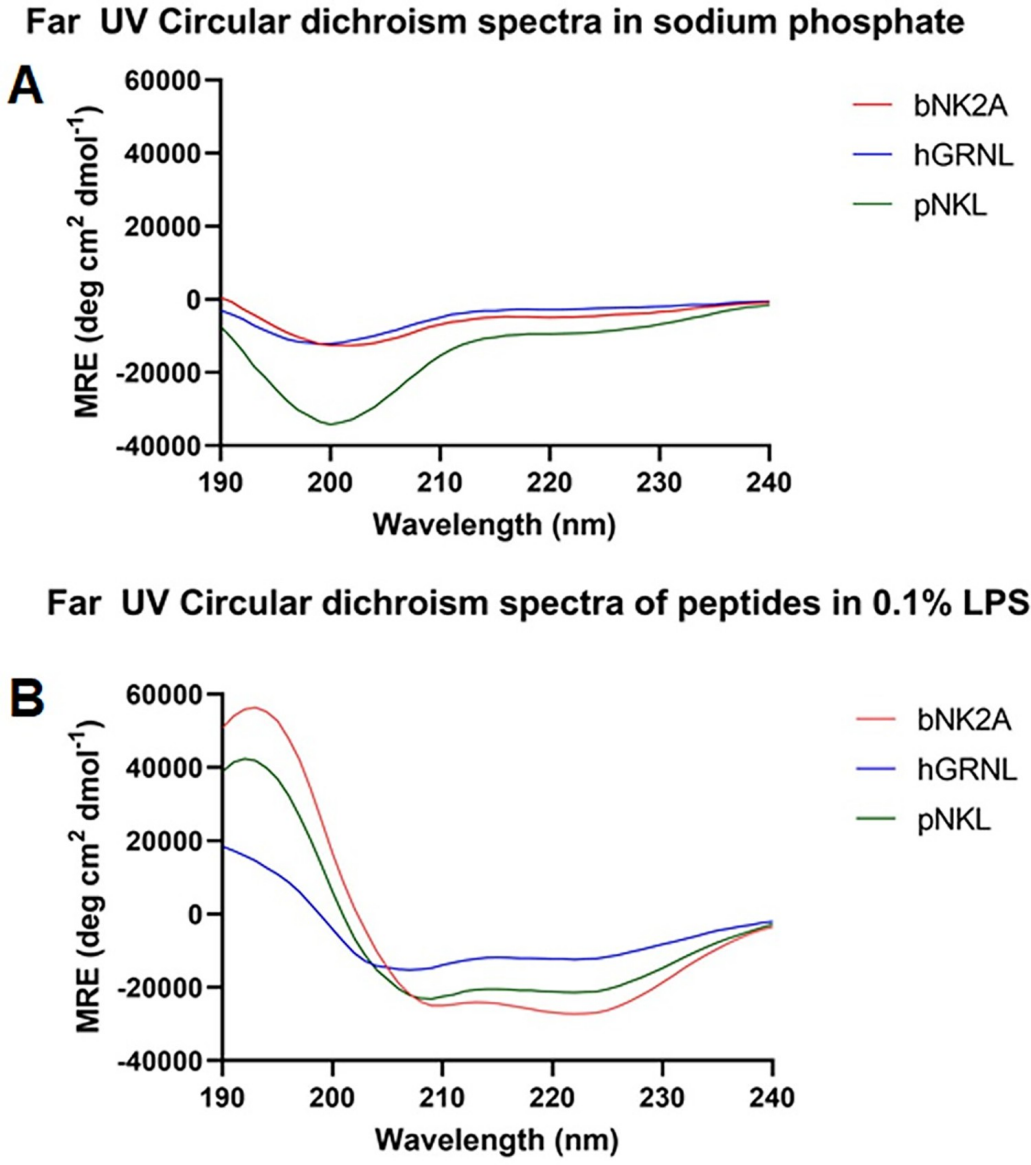
Circular dichroism (CD) spectrometry analyses of hGRNL, bNK2A, and pNKL peptides. CD spectra of all three AMPs (20 μM final concentrations) were determined in the aqueous buffer (A; 10 mM sodium phosphate) or LPS micelles (B; 0.1% (w/v). CD spectra were measured at room temperature using Jasco J-815 CD spectrometer and measurements were taken every 0.2nm from 250 to 190 nm wavelengths. Following background subtraction, raw data in millidegrees was converted to mean residue ellipticity (MRE). Average spectra with six accumulations after baseline correction for each AMPs are shown.

Circular dichroism spectra in buffer (NaPB) and anionic micelles (0.1% LPS (v/v) in NaPB) are shown in Fig 1. In NaPB, the spectra are consistent with a predominantly disordered structure (or random coil; minima near 200 nm) for all peptides while in LPS all peptides are consistent with a predominantly α-helical conformation (maxima near 192 nm and double minima near 208 nm and 222 nm). This observation suggests that rapid conformational changes of all three peptides upon binding with negatively charged LPS. hGRNL showed the lowest α-helical content while bNK2A showed the highest α-helical content. The highest β-turn, β-sheet and disordered contents was found in hGRNL as compared to bNK2A and pNKL. The results of CD secondary structural deconvolution as described in the materials and methods are summarized in Table 1.

**Table 1:** Percentages of estimated each type of secondary structure as determined by far UV circular dichroism for each peptide in the indicated solvent (LPS-free and LPS bound states).

Solvent	Peptide	α-helices	Turn	β-sheet	Disordered
Sodium phosphate buffer	hGRNL	10.3	24.8	29.6	35.4
	bNK2A	15.4	23.9	25.7	35.0
	pNKL	25.5	29.3	5.7	39.5
LPS	hGRNL	37.7	19.2	8.7	29.0
	bNK2A	78.7	7.9	0.9	12.4
	pNKL	67.2	13.6	2.3	17

### Hemolytic activity of granulysin and NK-lysins

**Fig 2:**
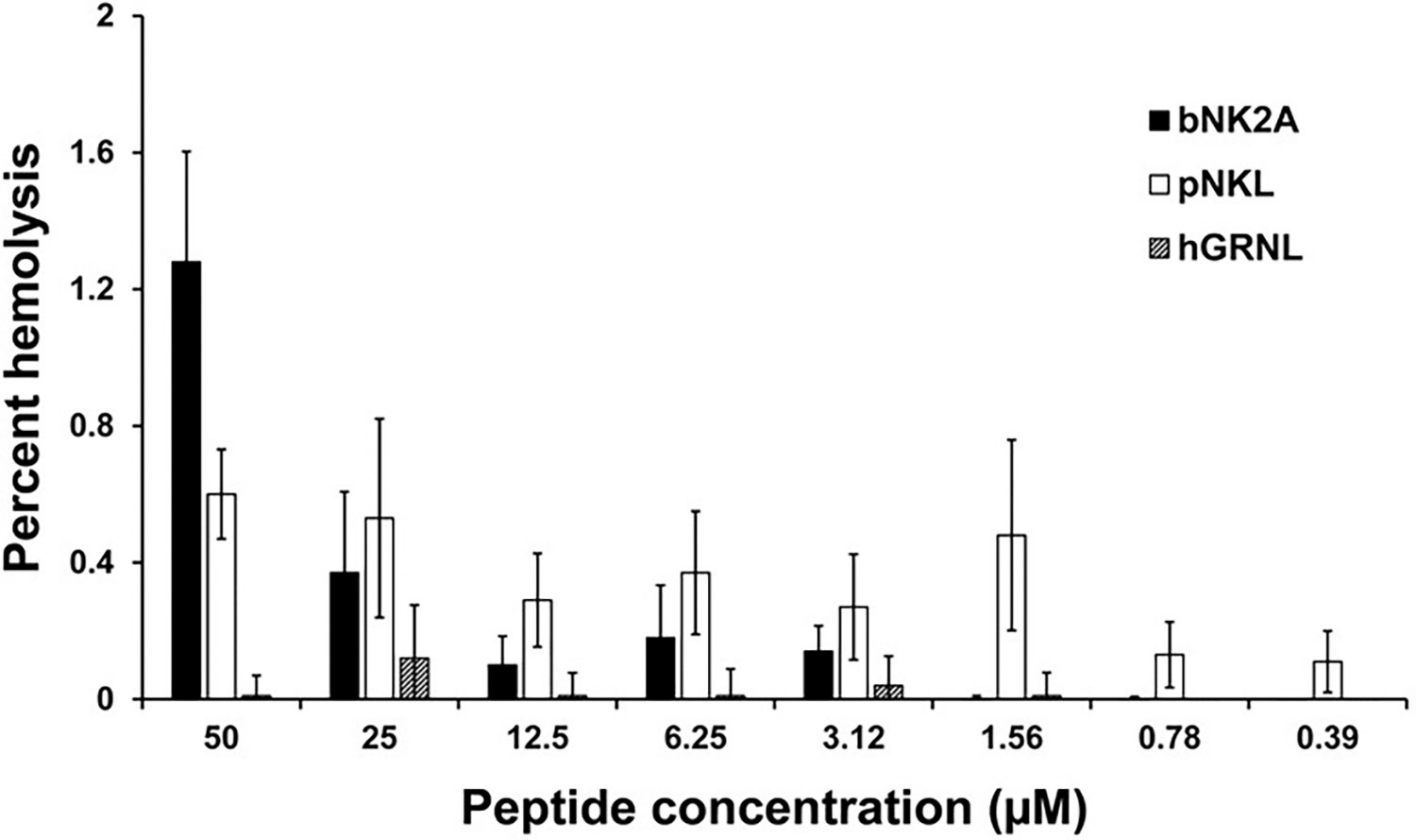
Hemolytic activities of hGRNL, bNK2A, and pNKL peptides on cattle red blood cells (RBCs). The cattle RBCs resuspended in PBS (2.5% hematocrit) were incubated with serially diluted three AMPs (two-fold dilution 50 to 0.39 μM) at 37°C in a humidified atmosphere for 60 min in two independent experiments. Percent hemolysis with SEM (y-axis) for each peptide concentration tested (x-axis) is depicted in the graph.

Hemolytic activity assay is the most widely used *in vitro* assay to measure the toxicity of AMPs and we have used this assay extensively to evaluate toxicity of bovine NK-lysin peptides [16, 26]. Therefore, to test the toxicity of hGRNL, bNK2A and pNKL in this study, we performed the hemolytic assay by incubating cattle RBCs with the AMPs and measured released hemoglobin by spectrophotometry. As expected, RBCs incubated with DPBS (negative control) showed only a minimal hemolysis (spontaneous) whereas, RBCs incubated with Triton X-100 (positive control) displayed complete hemolysis. All three AMPs showed very low hemolysis (<2%) even at the highest peptide concentration tested (50 μM; Fig 2).

### Antimicrobial activity of granulysin and NK-lysins on O157

We used chemically synthesized 29-30-mer peptides corresponding to the functional region (helix2-loop-helix3) of hGRNL, bNK2A and pNKL to determine their effectiveness against O157 strains. The most effective AMP against both O157 strains 86–24 and EDL933 in MHB was pNKL with an MIC and MBC of 6.25 μM compared to 12.5–25 μM for bNK2A and 200 μM for hGRNL (Table 2). The MBC (a 3-log reduction in growth or 99.9% killing) matched with the MIC of each peptide against the O157 strains. Based on these results, hGRNL was the least effective among the three AMPs, while pNKL was the optimal peptide after 20 h of treatment at 37˚C. As expected, both O157 strains were highly sensitive to the antimicrobial activity of antibiotics tetracycline (MIC/MBC: 0.5-1/2 μg/mL) and ciprofloxacin (MIC/MBC: <0.016/<0.016 μg/mL).

**Table 2:** Minimum inhibitory (MIC) and minimum bactericidal concentrations (MBC) of hGRNL, bNK2A, and pNKL on O157 strains 86–24 and EDL933.

**Strain 86–24**								
	Exp 1	Exp 2	Exp 3	Final MIC	Exp 1	Exp 2	Exp 3	Final MBC
hGRNL^a^	-	-	200	200	-	-	200	200
bNK2A^a^	12.5	12.5	12.5	12.5	12.5	25	12.5	12.5
pNKL^a^	6.25	6.25	6.25	6.25	6.25	6.25	6.25	6.25
Tetracycline^b^	-	0.5	0.5	0.5	-	2	2	2
Ciprofloxacin^b^	-	<0.016	<0.016	<0.016	-	<0.016	<0.016	<0.016
**Strain EDL933**								
	Exp 1	Exp 2	Exp 3	Final MIC	Exp 1	Exp 2	Exp 3	Final MBC
hGRNL*	-	-	200	200	-	-	200	200
bNK2A*	25	25	25	25	25	25	12.5	25
pNKL*	6.25	12.5	6.25	6.25	6.25	12.5	6.25	6.25
Tetracycline^#^	-	1	1	0.5	-	2	2	2
Ciprofloxacin^#^	-	<0.016	<0.016	<0.016	-	<0.016	<0.016	<0.016

^a^Peptide concentrations = μM

^b^Antibiotic concentrations: μg/mL

hGRNL was less effective against O157 strains than bNK2A and pNKL in MHB. It has been previously reported that antimicrobial activity of hGRNL can get inhibited at higher concentrations of cations [37]. Since cation-adjusted MHB has relatively high levels of divalent cations (Ca^2+^ and Mg^2+^), antimicrobial activity of hGRNL in MHB was compared with NaPB against O157 strain EDL933. A significant difference in the concentration of hGRNL needed to cause a 3-log reduction in O157 viability was observed between the two mediums following 3 h incubation (Table 3). The bactericidal activity of hGRNL in MHB was observed at 200 μM compared to 0.781 μM in NaPB, suggesting that as has been previously reported [37], divalent cations present in MHB interfere with the antimicrobial properties of hGRNL. In comparison, a slightly reduced bactericidal activity for ciprofloxacin was noticed in NaPB compared to MHB.

**Table 3:** Comparative analysis of the antimicrobial activity of hGRNL, bNK2A, and pNKL against O157 strain EDL933 in Müeller-Hinton broth (MHB) or sodium phosphate buffer (NaPB).

**MHB**			
	Exp 1	Exp 2	Average bactericidal concentration
hGRNL^a^	200	200	200
Ciprofloxacin^b^	<0.016	<0.016	<0.016
**NaPB**			
	Exp 1	Exp 2	Average bactericidal concentration
hGRNL^a^	0.781	0.781	0.781
bNK2A^a^	0.781	0.390	0.781
pNKL^a^	0.781	0.390	0.781
Ciprofloxacin^b^	0.5	0.5	0.5

^a^Peptide concentrations = μM

^b^Antibiotic concentration: μg/mL

We then sought to see if there would be a difference in the activity of pNKL and bNK2A against O157 strain EDL933 in NaPB, similar to what was observed for hGRNL. Both AMPs were tested in NaPB for 3 h under the same conditions, and regardless of peptide source, 100% bacterial killing was observed at 0.781 μM (Table 3). These results suggest that the cations present in MHB have an interfering mechanism against the antimicrobial activities of all three AMPs, especially hGRNL, as the 100% bacterial killing was achieved with NaPB at 0.781 μM concentration as compared to MIC/MBC of 200 μM in MHB.

### Propidium uptake (PI) uptake assay

**Fig 3:**
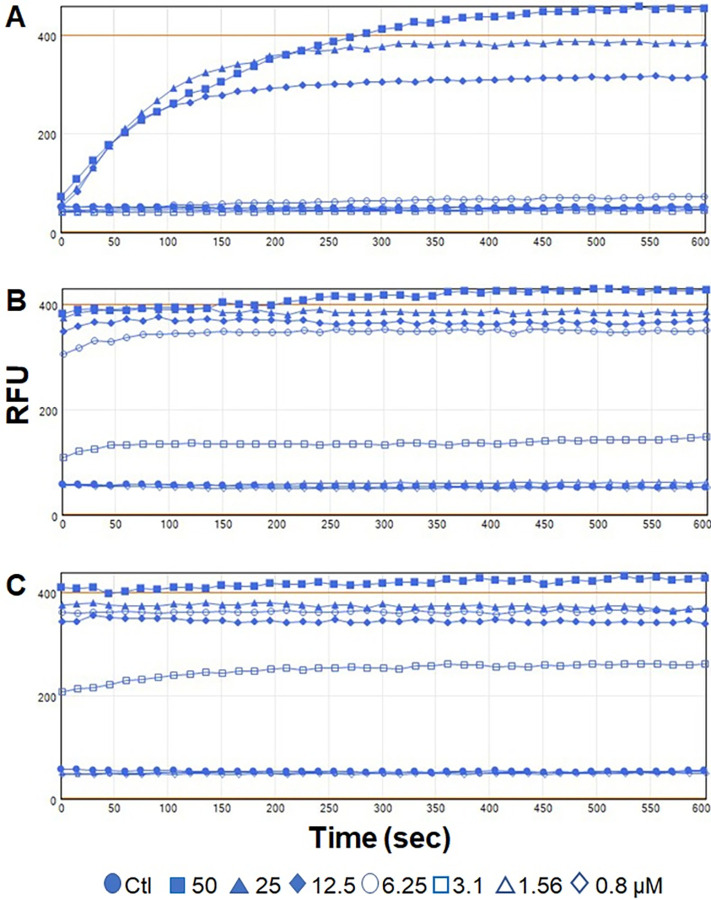
Evaluation of propidium iodide (PI) uptake by O157 following incubation with hGRNL, bNK2A, and pNKL peptides. O157 strain EDL933 was preincubated with PI (3 μM final concentration) for 5 min followed by incubation with serially diluted (A) hGRNL, (B) bNK2A, or (C) pNKL (two-fold dilution of 50 to 0.8 μM). PI uptake fluorescence signals (relative fluorescence units, RFU) were immediately measured using a fluorescent microplate reader with excitation and emission at 530 nm and 620 nm wavelengths, respectively. O157 strain EDL933 incubated with NaPB was used as a negative control. Results of one representative experiment out of three are shown.

To assess damage to bacterial membranes and to determine the kinetics of *E*. *coli* killing by three AMPs, PI uptake assay was performed in a dose- and time-dependent manner [26]. PI is a (red) fluorescent cell impermeant dye which can only bind with DNA and RNA of dead cells or cells with compromised membranes since PI is excluded from live cells. Three AMPs were tested at 0.9–50 μM final concentrations on O157 strain EDL933. As expected, three AMPs showed increased PI uptake signals indicating peptide-mediated damage to bacterial membranes leading to cell death (Fig 3). Both bNK2A and pNKL showed faster killing kinetics and increased PI uptake signals were observed at ≥3.2 μM concentration immediately after the incubation (Fig 3). Furthermore, PI signal intensity remained steady during 10 min incubation suggesting maximum damage to bacterial membranes occurred immediately following bacteria-peptide interactions. hGRNL also showed faster killing kinetics but the maximum PI uptake signal was observed at ~150–400 sec into the incubation. Unlike bNK2A and pNKL, increased PI uptake signal for hGRNL was visible at >12.5 μM concentration (Fig 3). Bacteria in the control sample showed no PI uptake signal.

### Transmission election microscopy (TEM)

**Fig 4:**
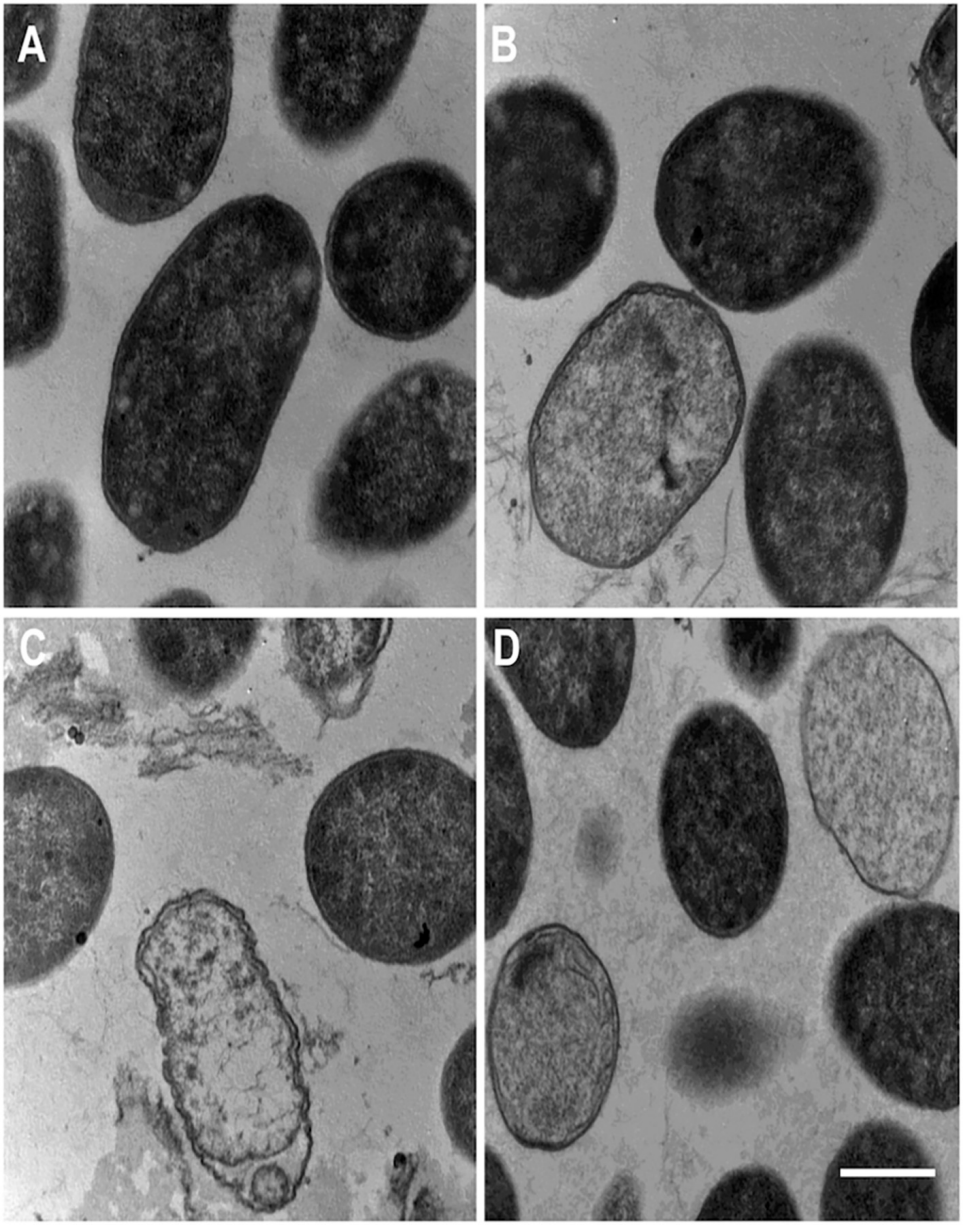
Transmission electron microscopic (TEM) visualization of ultrastructural changes to O157 membranes following incubation with hGRNL, bNK2A, and pNKL peptides. O157 strain EDL933 was incubated with the three AMPs (20 μM final concentration) at 37°C for 15 min and TEM was performed as described in the methods. O157 incubated with aqueous buffer (panel A; 30,000 × magnification) show bacteria with intact membranes. O157 incubated with hGRNL, bNK2A, and pNKL (panels B, C, D; 30,000 × magnification) show damage to outer LPS and inner cytoplasmic membranes. Results of one representative experiment out of three are shown. Scale bar = 500 nm.

Findings from this study (Fig 3) and previous PI uptake assays [16, 26] strongly suggest the increased PI fluorescent signals following incubation of O157 with three AMPs were due to the damages to membranes. It is known that hGRNL and NK-lysins induce pore formation of the membranes leading to bacterial death [16, 18, 26]. However, thus far, no studies have comparatively analyzed the ultrastructural changes to bacterial membranes in general and especially in O157, following incubation with hGRNL and NK-lysins. Therefore, to visualize ultrastructural changes to O157 membranes and compare such damages, TEM was performed. Outer LPS and inner cytoplasmic membranes of O157 incubated with NaPB (control) were intact, maintained bacilli-shape, and the cytosols were electron dense indicating bacteria were alive (Fig 4A). Furthermore, periplasmic spaces of control O157 was thin and uniform. In contrast, O157 incubated with hGRNL (Fig 4B), bNK2A (Fig 4C) and pNKL (Fig 4D) showed extensive damage to both outer LPS and inner cytoplasmic membranes. Electron-lucent cytosols were only visible in AMPs incubated O157 suggesting the leakage of cytoplasmic contents following damage to membranes. Additionally, AMPs treated O157 also showed wavy cytoplasmic membranes (Fig 4B amd 4C) and wider periplasmic spaces (especially with bNK2A, Fig 4C). These findings confirmed the pore formation properties of hGRNL, bNK2A and pNKL.

### Immunoassay for Shiga toxin expression

**Fig 5:**
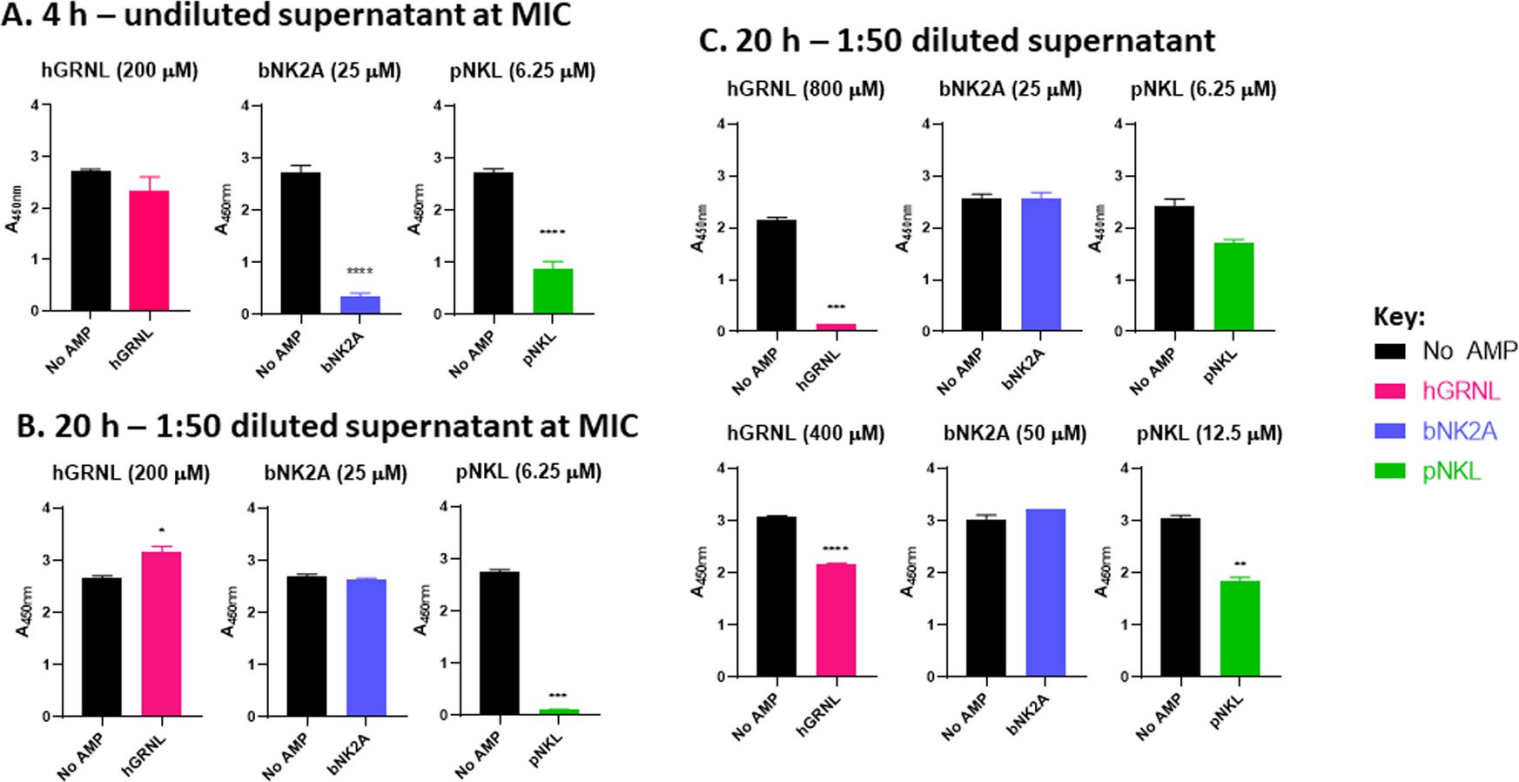
Shiga toxin expression in O157 following treatment with hGRNL, bNK2A, and pNKL peptides. Panel A: 4 h- undiluted supernatant at minimum inhibitory concentration (MIC). Log-phase O157 strain EDL933 cultures were incubated with the three antimicrobial peptides (AMPs) at respective minimum inhibitory concentrations. Panel B: 20 h- 1:50 diluted supernatant at MIC. Stationary-phase O157 strain EDL933 cultures were incubated with human granulysin, bovine and porcine NK-lysins at respective MICs. Panel C: 20 h- 1:50 diluted supernatant. Stationary-phase O157 strain EDL933 cultures were incubated with hGRNL (400–800 μM), bNK2A (25–50 μM), or pNKL (6.25–12.5 μM). Supernatants from three like wells were pooled to set up ELISAs and O157 with no AMP was included as a control. Average OD_450nm_ readings with standard error of mean are shown along the y-axis and displayed on the x-axis is O157 incubated with or without peptide. Unpaired *t*-tests were used to calculate the significance between samples of O157 without peptide and O157 with peptide, incubated for 4 h undiluted (n = 4) or 20 h 1:50 diluted (n = 2). * *p* = 0.0468; ** *p* = 0.0033; *** *p* = 0.0004; **** *p* <0.0001.

bNK2A and pNKL peptides at their respective MICs were non-inducing for Shiga toxin, as there was no additional stimulation after 4 h and 20 h incubation in MHB. Supernatants of both log- and stationary-phase O157 strain EDL933 cultures were tested for Shiga toxin production after treatment with hGRNL, bNK2A, or pNKL in an EHEC-ELISA. Log-phase cultures are important for testing toxin production, as any change in Stx induction in actively growing bacteria should be visible within 4 h. The use of stationary-phase cultures was included to match the conditions in the susceptibility assays, matching CLSI guidelines. Supernatants after 4 h of treatment were diluted using doubling dilutions to 1:256 and those after 20 h were diluted up to 1:1000, as we anticipated stationary-phase O157 cultures to accumulate more protein than log-phase cultures. Detection of Shiga toxin was observed in all cultures, regardless of treatment, after both 4 and 20 h. Viable O157 counts after 4 h of treatment at MIC were lower than cultures without AMP, irrespective of peptide (S1A Table). Optical density (OD_450nm_) readings for cultures treated with any of the three peptides (undiluted supernatants) were less than that of non-treated cultures after 4 h, with a significant decrease in OD in O157 incubated with bNK2A (p < 0.0001) or pNKL (p < 0.0001) (Fig 5A). Between the three peptides, bNK2A and pNKL had lower OD_450nm_ readings than hGRNL after 4 h (S1B Table).

However, OD readings of O157 cultures treated with hGRNL at MIC following 20 h incubation were significantly higher than cultures with no AMP (p = 0.0468) and those with pNKL at MIC were significantly lower than no AMP cultures (p = 0.0001), both supernatants diluted 1:50 (Fig 5B). Upon storage of reconstituted hGRNL and NK-lysins, a decrease in bactericidal activity was observed for all AMPs after 20 h incubation (S1A Table). Therefore, freshly dissolved peptides (in NaPB) were used in subsequent assays and multiple concentrations of peptides were evaluated. O157-hGRNL supernatants diluted 1:50 were significantly less than no AMP cultures at both 400 μM (p < 0.0001) and 800 μM (p = 0.0004), as were those treated with pNKL at 12.5 μM (p = 0.0033) (Fig 5C). OD_450nm_ readings of O157-bNK2A treated cultures were similar to no AMP cultures (S2B Table). These findings indicate the pNKL and bNK2A AMPs do not induce Shiga toxin production after both 4 h and 20 h, while hGRNL at MIC (200 μM) appears to induce toxin production after 20 h of treatment. Further, over time, pNKL appears to continue to suppress Stx induction while the effect of bNK2A on Stx production early on wears off over time.

## Discussion

The efficacy of three AMPs, hGRNL, bNK2A, and pNKL, was tested *in vitro* against two O157 strains for potential development into novel pre-harvest, non-antibiotic agents to reduce STEC shedding in cattle. CD spectroscopy analysis revealed the three peptides have a predominantly α-helical conformation in the presence of LPS and hGRNL had the highest disordered contents. All peptides displayed minimal cytotoxicity against cattle red blood cells at concentrations up to 50 μM, and bNK2A and pNKL at MIC did not further stimulate Stx production in O157 after 4 h and 20 h of treatment. Findings from peptide susceptibility assays, in accordance with the CLSI guidelines, revealed the MICs against O157 matched the MBCs of hGRNL (200 μM), bNK2A (12.5–25 μM), and pNKL (6.25 μM). Further, bNK2A and pNKL displayed the fastest killing kinetics and membrane damage to O157, as demonstrated by propidium iodide uptake assays, and required the lowest concentrations to cause damage to bacterial membranes upon TEM analysis. These findings lend support for further analysis of bNK2A and pNKL peptides as non-antibiotic agents against O157, as STEC continues to make a large, global impact on the food industry and on human health.

The U.S. Department of Agriculture-Food Safety and Inspection Service (USDA-FSIS) describes current (2014) pre-harvest management controls and intervention guidelines to reduce STEC shedding in cattle. Current pre-harvest research practices range from exposure (clean and dry bedding; maintain closed herds; avoiding cross contamination between animals during transportation), exclusion (water and feed additives such as antibiotics, probiotics, seaweed extract; grain versus forage diets; fasting prior to slaughter), and direct (hide washing; live animal treatments such as bacteriophages, vaccines, and competitive exclusion) reduction practices [38]. Some of these practices are currently being researched for efficacy, while others are not easily produced or have insufficient data. As such, additional safe and effective intervention options should be explored to control O157 shedding in cattle and thereby reduce food contamination.

Based on the established previous success of bovine NK-lysin peptides against foodborne pathogen *Salmonella* [16], bNK2A, hGRNL, and pNKL peptides corresponding to the functional region helix2-loop-helix3 were selected as potential anti-STEC agents. Human granulysin, bovine, and porcine NK-lysins have shown to be effective against Gram-negative bacteria [17, 19]. These peptides are part of the innate immune response, produced by cytotoxic T lymphocytes and natural killer cells [20–22], and are similar in terms of structure and function. However, we observed structural differences among the three peptides upon CD spectrometry evaluation. bNK2A displayed the highest α-helical content, while hGRNL showed the lowest but displayed the highest β-turn, β-sheet and disordered contents compared to the other two AMPs (Fig 1, Table 1). Based on these findings, we expected the hGRNL peptide to have minimal activity compared to bNK2A and pNKL peptides.

Antimicrobial peptide killing assays are typically performed in bacteria resuspended in bacterial broth media [18, 20] or buffer (NaPB, KPB or PBS) [24, 26] along with AMPs dissolved in buffer (NaPB, KPB, or PBS) [16, 20, 24] or water [19]. It is our understanding that hGRNL assays are typically performed in NaPB [37, 39]. It has previously been shown that hGRNL is sensitive to normal physiological concentrations of NaCl (150 mM) and MgCl_2_ (1.5 mM) [37]. Therefore, higher MIC/MBC values observed with hGRNL (200 μM) as compared to bNK2A and pNKL is most likely attributed to the presence of divalent cations in cation-adjusted MHB (Table 2). Although lower peptide concentrations of bNK2A (MIC/MBC = 12.5–25 μM) and pNKL (MIC/MBC = 6.25 μM) were effective against O157 in the microbroth dilution assay (in MHB medium), much lower peptide concentrations of all three AMPs (0.781 μM) were needed to kill 100% of O157 when the assay was performed in NaPB for 3 hrs (Table 3). This observation suggests that cations can show modest interference of bactericidal activity of bNK2A and pNKL as compared to hGRNL. Taken together, these findings suggest that pNKL and bNK2A are better peptides for future *in vivo* studies.

These observations are further supported by results from both PI uptake and TEM. O157 incubated with peptide displayed an increase in PI uptake signal, indicating damage to the membranes following bacteria-peptide interactions, as the PI dye binds only to DNA or RNA of dead or compromised cells. Compared to bNK2A and pNKL (>3.2 μM), a higher concentration (>12.5 μM) and longer incubation time (~150–400 sec versus immediately following incubation) were required to damage O157 membranes with hGRNL (Fig 3). This could be due to the higher number of disordered contents and β-turn, β-sheet in hGRNL than in bNK2A and pNKL, observed from CD spectroscopy. To damage bacterial membranes, these three AMPs are known to induce pore formation upon interaction with bacterial cells [16, 18, 26] and the properties appear to be similar. TEM analysis of O157 incubated with hGRNL, bNK2A, or pNKL confirmed the damage to the bacterial membrane was caused by pore formation mechanisms of the hGRNL and NK-lysins (Fig 4). Expected AMP membranolysis mechanisms (barrel-stave, toroidal-pore, carpet, or aggregate model) [14, 15] were further supported by TEM, as wavy cytoplasmic membranes, wider periplasmic spaces, and electron-lucent cytosols were observed only in AMP-treated O157, suggesting cytoplasmic content leakage.

Furthermore, NK-lysins did not appear to be toxic to mammals, as inferred from cattle red blood cell hemolysis assay, Shiga toxin production evaluations, and previous mouse bioassay [40]. The direct toxic effect was evaluated with the hemolytic assay in which all three AMPs showed low hemolysis of cattle RBCs (<2%), even at 50 μM (Fig 2). The indirect toxic effect was determined with the ELISA evaluating the production of Shiga toxins (Stx) by two different growth phase cultures of O157. Log-phase (4 h) cultures, in which bacteria are actively growing, are important for testing toxin production, as any change in Stx would be visible within the timeframe. Stationary-phase (20 h) cultures, at which point the bacteria are dying, were included in analysis to match the conditions of our susceptibility assays and the CLSI guidelines. We anticipated more protein accumulation with stationary-phase O157 than with log-phase cultures, and thus, used different sets of dilutions to dilute Stx in the ELISA. At MIC, the bNK2A and pNKL peptides in MHB did not further stimulate Stx production by O157, post -4 h or -20 h incubation (Fig 5A and 5B). Further, the suppression of Stx after pNKL treatment (at MIC) remained over time, as observed with both 4 h and 20 h, but the suppression effect bNK2A displayed at 4 h wore off over time, as after 20 h, with Stx production being similar to that of O157 without AMP. This suggests that the pNKL peptide was more stable and effective than bNK2A. There was a significant increase in Stx production when O157 was incubated with hGRNL at MIC after 20 h but not 4 h. Interestingly, when higher concentrations of hGRNL were applied (400 and 800 μM), after 20 h, Stx production was significantly lower than cultures with no AMP (Fig 5C).

The MIC and MBC of hGRNL and NK-lysins were identical in initial susceptibility trials, with the use of freshly reconstituted AMPs. pNKL was the most effective peptide out of the three AMPs tested against O157 strains 86–24 and EDL933 in MHB after 20 h, at a concentration of 6.25 μM, with bNK2A at 12.5–25 μM, and hGRNL the least effective at 200 μM (Table 2). Because previous studies have shown that the antimicrobial activity of hGRNL can be inhibited in the presence of cations [37], we next sought to see if the divalent cations present in MHB were having such an inhibitory effect on hGRNL. There was indeed a large decrease in bactericidal concentration of hGRNL against O157 strain EDL933 after 3 h when tested in NaPB. Compared to 200 μM in MHB, the concentration was greatly reduced to 0.781 μM in NaPB (Table 3), suggesting that the cations in MHB do interfere with antimicrobial activity of hGRNL. We achieved the same results for bNK2A and pNKL when tested in NaPB under the same conditions- 100% of O157 were killed at 0.781 μM, suggesting cations have an interfering effect with all peptides tested, albeit to a lesser extent for bNK2A and pNKL. However, NaPB is not representative of physiological conditions *in vivo*, and therefore does not mimic the types of conditions the peptides would encounter upon application.

The peptide concentrations needed to elicit a bactericidal effect against both O157 strains remained consistent in initial susceptibility trials with the use of fresh, reconstituted AMPs. However, in subsequent assays (4 h and 20 h ELISA) using previously reconstituted peptides stored at -80˚C, a loss of peptide efficacy was observed (S1A Table). Higher concentrations of hGRNL (800 μM) and pNKL (12.5 μM) were needed to achieve a bactericidal effect on O157, while concentrations up to 50 μM for bNK2A had no bactericidal effect (S2A Table). These results indicate reconstituted (DPBS or NaPB) peptides become labile during long-term storage. Extended storage of peptides in aqueous solution can result in spontaneous chemical modification or aggregation and thus result in a reduction or loss of activity. Even though the AMPs were modified at the N’ and C’ terminals to prevent enzymatic degradation upon storage, unknown structural or amino acid alterations may have interfered with peptide activity. Additional experiments to minimize such alterations are ongoing.

Overall, the results from this study indicate that out of the three peptides evaluated, pNKL has the most potential for development into a non-antibiotic, anti-STEC agent. Future studies evaluating the bactericidal effect of pNKL against other O157 and non-O157 isolates are being planned.

## Conclusions

Out of the three AMPs evaluated, pNKL shows the most promise to be developed into a non-antibiotic pre-harvest agent to reduce O157 shedding by cattle. Through various assays, pNKL caused little-to-no toxicity to the host, was the most stable during storage, and required the lowest concentration to elicit a bactericidal effect against O157.

## Supporting information

S1 Table Viable counts (A) and EHEC-ELISA OD_450nm_ readings (B) of O157 strain EDL933 log-phase (4 h) and stationary-phase (20h) cultures, post-incubation with hGRNL, bNK2A, and pNKL at MIC in Müeller-Hinton broth.
(DOCX)

S2 Table Viable counts (A) and EHEC-ELISA OD_450nm_ readings (B) of O157 strain EDL933 stationary-phase (20 h) cultures, post-incubation with hGRNL (400–800 μM), bNK2A (25–50 μM), and pNKL (6.25–12.5 μM) in Müeller-Hinton broth.
(DOCX)
