# Supplementary material for: Comparative evaluation of antimicrobial activity of human granulysin, bovine and porcine NK-lysins against Shiga toxin-producing Escherichia coli O157:H7
Source: PLoS One. 2023 Sep 28;18(9):e0292234. doi: 10.1371/journal.pone.0292234 (PMC10538649; doi:10.1371/journal.pone.0292234)
Supplement: S1 Table — Viable counts (A) and EHEC-ELISA OD450nm readings (B) of O157 strain EDL933 log-phase (4 h) and stationary-phase (20h) cultures, post-incubation with hGRNL, bNK2A, and pNKL at MIC in Müeller-Hinton broth. (DOCX) [file pone.0292234.s001.docx]

**S1 Table.** Viable counts and EHEC-ELISA OD_450nm_ readings of O157 strain EDL933 log-phase (4 h) and stationary-phase (20h) cultures, post-incubation with hGRNL, bNK2A, and pNKL at MIC in Müeller-Hinton broth.

**A. Viable counts.**

|  | **Viable count (CFU/mL)^a^** | | |
| --- | --- | --- | --- |
| **AMP / concentration (μM)** | **Assay A1^b^** | **Assay A2^b^** | **Assay B1^c^** |
| hGRNL / 200 | 4.00x10^3^ | 1.10x10^5^ | 3.71x10^6^ |
| bNK2A / 25 | 1.30x10^4^ | 1.67x10^2^ | 2.22x10^6^ |
| pNKL / 6.25 | 3.67x10^4^ | 6.67x10^5^ | NG |
| No AMP | Not plated | 2.33x10^6^ | 2.16x10^6^ |

^a^ Cultures from three like wells were pooled to determine viable counts.
^b^ Viable counts from log-phase (4h) cultures.
^c^ Viable counts from stationary-phase (20h) cultures.

**B. OD_450nm_ readings from the EHEC-ELISA used to determine Shiga toxin production.**

|  | **hGRNL** | | | | | | **bNK2A** | | | | | | **pNKL** | | | | | |
| --- | --- | --- | --- | --- | --- | --- | --- | --- | --- | --- | --- | --- | --- | --- | --- | --- | --- | --- |
|  | **Assay A1^c^** | | **Assay A2^c^** | | | **Mean ±SEM** | **Assay A1^c^** | | **Assay A2^c^** | | | **Mean ± SEM** | **Assay A1^c^** | | **Assay A2^c^** | | | **Mean ± SEM** |
| **AMP undiluted^a^** | 1.787 | 1.886 | 2.711 | | 2.901 | **2.321±0.2833** | 0.432 | 0.484 | 0.234 | | 0.247 | **0.3493±0.0637** | 0.661 | 0.614 | 1.103 | | 1.127 | **0.8763±0.1383** |
| **No AMP undiluted^a^** | 2.647 | 2.831 | 2.640 | | 2.729 | **2.712±0.0446** | 2.955 | 2.868 | 2.303 | | 2.712 | **2.710±0.1445** | 2.692 | 2.600 | 2.667 | | 2.927 | **2.722±0.0712** |
| ***p* value^b^ (AMP vs no AMP)** | 0.2222 | | | | | | <0.0001 | | | | | | <0.0001 | | | | | |
|  | **hGRNL** | | | | | | **bNK2A** | | | | | | **pNKL** | | | | | |
|  | **Assay B1^d^** | | | | | **Mean ±SEM** | **Assay B1^d^** | | | | | **Mean ± SEM** | **Assay B1^d^** | | | | | **Mean ± SEM** |
| **AMP diluted 1:50^a^** | 3.059 | | | 3.268 | | **3.164±0.1045** | 2.663 | | | 2.611 | | **2.637±0.0260** | 0.115 | | | 0.113 | | **0.1140±0.0010** |
| **No AMP diluted 1:50^a^** | 2.705 | | | 2.627 | | **2.666±0.0390** | 2.635 | | | 2.736 | | **2.686±0.0505** | 2.789 | | | 2.731 | | **2.760±0.0290** |
| ***p* value^b^ (AMP vs no AMP)** | 0.0468 | | | | | | 0.4831 | | | | | | 0.0001 | | | | | |

^a^ Supernatants from three like wells were pooled to set up the ELISAs.

^b^ Unpaired t-test was used to determine the *p* value.
^c^ OD_450nm_ readings from log-phase (4h) cultures.
^d^ OD_450nm_ readings from stationary-phase (20h) cultures.
