# Supplementary material for: Comparative evaluation of antimicrobial activity of human granulysin, bovine and porcine NK-lysins against Shiga toxin-producing Escherichia coli O157:H7
Source: PLoS One. 2023 Sep 28;18(9):e0292234. doi: 10.1371/journal.pone.0292234 (PMC10538649; doi:10.1371/journal.pone.0292234)
Supplement: S2 Table — Viable counts (A) and EHEC-ELISA OD450nm readings (B) of O157 strain EDL933 stationary-phase (20 h) cultures, post-incubation with hGRNL (400–800 μM), bNK2A (25–50 μM), and pNKL (6.25–12.5 μM) in Müeller-Hinton broth. (DOCX) [file pone.0292234.s002.docx]

**S2 Table.** Viable counts and EHEC-ELISA OD_450nm_ readings of O157 strain EDL933 stationary-phase (20 h) cultures, post-incubation with hGRNL (400-800 μM), bNK2A (25-50 μM), and pNKL (6.25-12.5 μM) in Müeller-Hinton broth.

**A. Viable counts.**

|  | **Assay C1^a^** | | **Assay C2^a^** | |
| --- | --- | --- | --- | --- |
| **AMP** | **AMP concentration (μM)** | **Average viable count (CFU/mL)** | **AMP concentration (μM)** | **Average viable count (CFU/mL)** |
| hGRNL | 800 | NG | 400 | 3.25x10^2^ |
| bNK2A | 25 | 1.96x10^8^ | 50 | 3.15x10^8^ |
| pNKL | 6.25 | 4.83x10^7^ | 12.5 | NG |
| No AMP | - | 1.55x10^8^ | - | 1.38x10^8^ |

^a^ Cultures from each well were plated to determine viable counts; average of two are shown.

**B. OD_450nm_ readings from the EHEC-ELISA used to determine Shiga toxin production.**

|  | **hGRNL** | | | **bNK2A** | | | **pNKL** | | |
| --- | --- | --- | --- | --- | --- | --- | --- | --- | --- |
|  | **Assay C1** | | **Mean ± SEM** | **Assay C1** | | **Mean ± SEM** | **Assay C1** | | **Mean ± SEM** |
| **AMP diluted 1:50^a^** | 0.143 | 0.141 | 0.1420±0.0010 | 2.471 | 2.678 | 2.575±0.1035 | 1.631 | 1.774 | 1.703±0.0715 |
| **No AMP diluted 1:50^a^** | 2.121 | 2.197 | 2.159±0.0380 | 2.645 | 2.494 | 2.570±0.0755 | 2.552 | 2.257 | 2.405±0.1475 |
| ***p* value^b^ (AMP vs no AMP)** | 0.0004 | | | 0.9724 | | | 0.0504 | | |
|  | **Assay C2** | | **Mean ± SEM** | **Assay C2** | | **Mean ± SEM** | **AssayC2** | | **Mean ± SEM** |
| **AMP diluted 1:50^a^** | 2.181 | 2.170 | 2.176±0.0055 | 3.219 | 3.218 | 3.219±0.0005 | 1.805 | 1.908 | 1.857±0.0515 |
| **No AMP diluted 1:50^a^** | 3.089 | 3.077 | 3.083±0.0060 | 2.947 | 3.104 | 3.026±0.0785 | 3.099 | 3.008 | 3.054±0.0455 |
| ***p* value^b^ (AMP vs no AMP)** | <0.0001 | | | 0.1332 | | | 0.0033 | | |

^a^ Supernatants from three like wells were pooled and diluted accordingly for use in the ELISA.

^b^ Unpaired t-test was used to determine the *p* value.
